# Modulating the gut microbiota ameliorates spontaneous seizures and cognitive deficits in rats with kainic acid-induced status epilepticus by inhibiting inflammation and oxidative stress

**DOI:** 10.3389/fnut.2022.985841

**Published:** 2022-08-29

**Authors:** Xue Wang, Chunyu Yang, Liu Yang, Yongbo Zhang

**Affiliations:** ^1^Department of Neurology, Beijing Friendship Hospital, Capital Medical University, Beijing, China; ^2^Department of Neurology, Dehui People's Hospital, Jilin, China

**Keywords:** epilepsy, gut dysbiosis, oxidative stress, inflammation, cognition

## Abstract

**Introduction:**

Epilepsy is a highly prevalent neurological disease whose treatment has always been challenging. Hence, it is crucial to explore the molecular mechanisms underlying epilepsy inhibition. Inflammation and oxidative stress are important pathophysiological changes in epilepsy that contribute to the development of spontaneous seizures and cognitive deficits. In recent years, altered gut microbiota composition was found to be involved in epilepsy, but the underlying mechanism remains unclear. Modulation of the gut microbiota showed a positive impact on the brain by regulating oxidative stress and inflammation. Hence, this study evaluated the effect of modulating gut dysbiosis by treating epileptic rats with prebiotics, probiotics, and synbiotics and investigated the underlying molecular mechanism.

**Materials and methods:**

Epileptic rat models were established by injecting 1 μl of kainic acid (KA, 0.4 μg/μl) into the right amygdalae. The rats were divided into Sham, KA, KA+prebiotic [inulin:1 g/kg body weight (bw)/day], KA+probiotics (10 × 10^9^cfu of each bacteria/kg, bw/day), and KA+synbiotic groups (1:1 mixture of prebiotics and probiotics). Seizures were monitored, and cognitive function was assessed in all rats. Biochemical indicators, namely, oxidative stress, DNA damage, glutamate levels, and inflammation markers, were also determined.

**Results:**

The KA-induced status epilepticus (SE) rats exhibited spontaneous seizures and cognitive deficits. This was accompanied by the activation of glial cells, the inflammatory response (IL-1 β, IL-6, and TNF-α), lipid peroxidation (MDA), DNA damage (8-OHdG), the release of glutamate, and a decline in total antioxidant ability (GSH). These changes were alleviated by partial treatment with prebiotics, probiotics, and synbiotics.

**Conclusion:**

Modulating gut dysbiosis ameliorates spontaneous seizures and cognitive deficits in rats with KA-induced status epilepticus. The underlying mechanism may potentially involve the inhibition of inflammation and oxidative stress.

## Introduction

Epilepsy is a highly prevalent neurological disease affecting millions of people worldwide (1). Patients with recurrent seizures usually have disorders in cognition, sleep, and neuropsychiatry, which result in a low quality-of-life (2–4). Despite various anti-seizure medications (ASMs) used in the clinical treatment of epilepsy, the seizures of 30% of patients cannot be well-controlled, leading to the development of refractory epilepsy (5). It is not sufficient to control seizures by using ASMs only; the ultimate goal of epilepsy treatment is to find a breakthrough in inhibiting its occurrence and development. Neuroinflammation and oxidative stress are important pathophysiological changes in epilepsy and are associated with an enhanced risk of developing the disease (6). Hence, targeting inflammation and oxidative stress are crucial therapeutic approaches to inhibit the development of epilepsy and its associated comorbidities.

The microbiota-gut-brain axis is gradually recognized to play an important role in the central nervous system. The bidirectional connection between the brain and gut is mediated by the immune system, enteric nervous system (ENS), vagus nerve, and microbial metabolites (7). Some studies have reported the presence of an altered gut microbiota composition in drug-resistant patients, suggesting that gut dysbiosis might be involved in the mechanism of epilepsy (8, 9). Host bacteria have been found to regulate the maturation and function of microglia and the microglia-mediated inflammatory response (10, 11). In addition, oxidative stress during seizures is mainly caused by NADPH oxidases, especially those expressed in activated microglia (12, 13). The effect of gut bacteria on regulating neuroinflammation and oxidative stress has been directly confirmed in models of Alzheimer's disease (14). Therefore, modulating the gut microbiome may be a potential intervention strategy to inhibit the development of epilepsy.

The addition of probiotics, prebiotics, and synbiotics can be used to modulate gut microbiota. Probiotics are live microorganisms that exert beneficial effects on body health by improving intestinal and immune homeostasis (15, 16). Lactobacilli and bifidobacteria are well-known probiotic strains that have shown positive effects on several neurological and psychological diseases (17–19). Prebiotics, regarded as non-digestible food fibers, can improve the health of the host by selectively increasing the growth and activity of gut microbes, especially *Lactobacillus* and *Bifidobacterium* (20). Synbiotics are a combination of prebiotics and probiotics, in which the prebiotic components are beneficial for the growth and metabolism of the probiotics (20). In this study, we aimed to investigate the effects of prebiotics, probiotics, and synbiotics on kainic acid-induced status epilepticus in rats and elucidate the underlying mechanism of their action.

## Materials and methods

### Animals

Male Wistar rats (Beijing Vital River Laboratory Animal Technology Co. Ltd, China) weighing 280–300 g were used in this study. All experimental rats were raised in a standard environment with a 12-h light/dark cycle, constant temperature (22 ± 2°C), and free access to water and chow. All procedures in the experiment followed guidelines on the care and use of laboratory animals by the Capital Medical University (China). The ethics approval for this study was obtained from the Animal Studies Subcommittee of Capital Medical University (China).

### Drug preparation

Kainic acid (KA; Abcam, Cambridge, UK) was used to induce status epilepticus brain insults. To create a working solution, 0.4 μg KA was dissolved in 1 μl phosphate-buffered saline (PBS). Inulin (1 g/kg bw/day; Chicory, Sigma-Aldrich, MO, USA) was used as the prebiotic supplement. Probiotics containing *Bifidobacterium* and *Lactobacillus* were provided by Shanxi Sciphar Natural Products Co., Ltd. Approximately 10 × 10^9^ cfu of each probiotic per kg bodyweight was used for treating the experimental rats. The synbiotics used were a 1:1 mixture of prebiotics and probiotics.

### Seizure induction and experimental groups

Rat epilepsy models were anesthetized and positioned in a stereotaxic frame. Then, 1 μl KA solution was injected into the right amygdalae (2.5 mm caudal to the bregma; 4.5 mm right of the midline; 8.5 mm from the surface of the skull) to establish status epilepticus (SE) insult. Only rats that reached the fifth seizure stage (21) and were maintained for 1 h were allowed in this study to ensure consistency of brain injury and minimize suffering. Diazepam (8 mg/kg; Sigma-Aldrich) was used to terminate the seizures.

Rats were randomly divided into five groups. The sham group was injected with 1 μl PBS in the right amygdala and was fed orally with 1 mL PBS per day. The KA group was injected with 1 μl KA solution into the right amygdala and orally fed with 1 ml PBS daily. The KA+prebiotic group was injected with 1 μl KA solution into the right amygdala and orally fed with 1 ml PBS containing calculated prebiotics per day. The KA+probiotics group was injected with 1 μl KA solution into the right amygdala and orally fed with 1 ml PBS containing calculated probiotics per day. At last, the KA+synbiotic group was injected with 1 μl KA solution into the right amygdala and orally fed with 1 mL PBS containing calculated synbiotics per day.

All the experimental rats were monitored *via* video for spontaneous seizures from days 1 to 28. Latency (the time from SE induction to the first seizure episode), frequency of spontaneous seizures, the duration of the seizures, and scores of the seizures were recorded and evaluated for analysis.

### Morris water maze (MWM) test

The experimental rats were tested for spatial learning and memory ability using an MWM 14 days following seizure induction. This experimental device included a circular tank (divided into four quadrants), a platform (placed 1 cm below the water surface in one quadrant), and visual cues (placed inside the pool). Data were tracked and monitored using the Viewer 2 tracking software (China).

The rats were trained for five consecutive days (four 90-s trials per day) in the water maze. They were required to find the hidden platform within 90 s and were placed on different starting points for each trial. If the rats could not complete the goal within 90 s, they were directed and positioned on the platform for 10 s. The time each rat spent reaching the platform was recorded as the escape latency, with 90 s being the maximum time. On the sixth day, the learning ability and memory of the rats were assessed with a 90-s trial. In this trial, the platform was removed, and all rats were positioned in the quadrant opposite to the previous target quadrant. The escape latency, number of platform crossings, and time each rat spent in the target quadrant were recorded.

### Brain sample collection

In total, 3 days following SE induction, all experimental rats were sacrificed, their hippocampal tissues were extracted, and placed in ice-cold saline. The hippocampal samples were blotted and weighed, and 100 mg of wet tissue was homogenized in 1 ml phosphate buffer (0.1 M, pH 7.4). The homogenate was centrifuged (3,000 rpm, 4°C, 15 min), and the supernatant was collected and stored at −80°C for biochemical assays.

### Detection of biochemical parameters

The presence of the nucleotide 8-hydroxy-2-deoxyguanosine (8-OhdG) is an indicator of DNA damage. The concentrations of 8-OHdG were detected using an 8-OHdG assay kit (Nanjing Jiancheng Biotechnology Co., Ltd., China). Glutamate is an important excitatory neurotransmitter in the central nervous system, which was measured by using a Glutamate measurement kit (Nanjing Jiancheng Biotechnology Co., Ltd.). Malondialdehyde (MDA), a marker of lipid peroxidation, was detected using a thiobarbituric acid (TBA)-based spectrophotometric assay kit (Nanjing Jiancheng Biotechnology Co., Ltd.). The level of intracellular reduced glutathione (GSH) was measured using a GSH assay kit according to the manufacturer's protocol (Nanjing Jiancheng Biotechnology Co., Ltd.).

Concentrations of interleukin (IL)-1β, IL-6, interferon (IFN)-γ, and tumor necrosis factor (TNF)-α in the hippocampus were detected using ELISA kits according to the manufacturer's protocols (Nanjing Jiancheng Biotechnology Co., Ltd.). Ionized calcium-binding adapter protein-1 (Iba-1) and glial fibrillary acidic protein (GFAP) represent the activation of microglia and astrocytes, respectively. The levels of Iba-1 (Shanghai Runyu Biotechnology Co., Ltd., China) and GFAP (provided by Shanghai Zeye Technology Co., Ltd., China) were measured using ELISA kits according to the respective manufacturer's instructions.

### Statistical analysis

All the experimental data were analyzed using SPSS statistical software (version 21.0) and GraphPad Prism software (version 7.0). The data are expressed as the mean ± standard deviation (SD). Differences between groups were compared using Student's *t*-test or repeated-measures ANOVA. Tukey's test was used as a *post-hoc* analysis for multiple comparisons. Statistical significance was set as follows: ^*^ *P* < 0.05, ^**^P < 0.01.

## Results

### Modulating the gut microbiota ameliorates spontaneous seizures in epileptic rats

**Figure 1:**
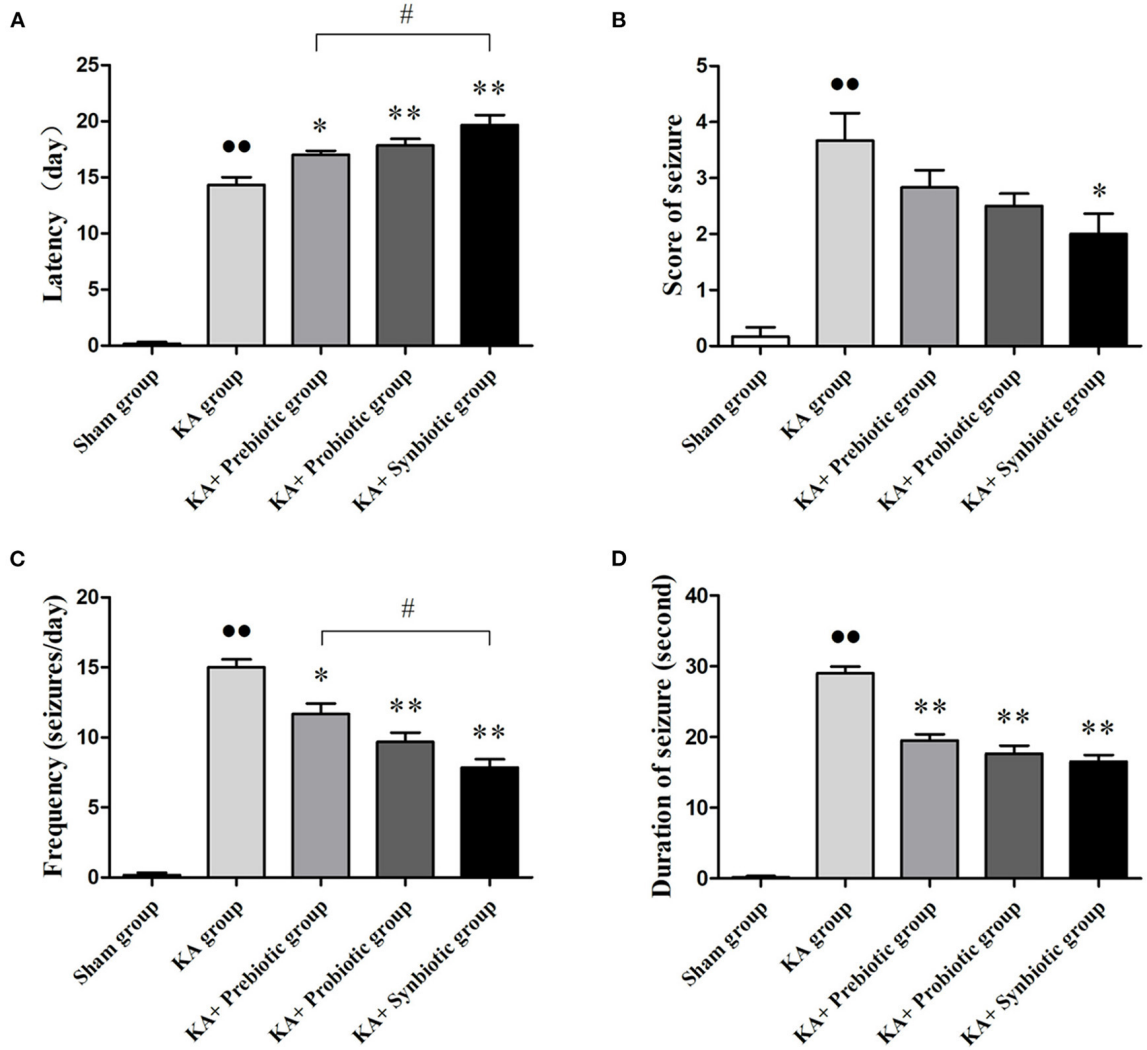
Effect of prebiotics, probiotics, and synbiotics on spontaneous seizures in rats following status epilepticus induction. **(A)** Latency. **(B)** Scores of seizures. **(C)** Frequency of seizures. **(D)** Duration of seizures. ∙∙ *P* < 0.01 vs. the Sham group, ^*^P < 0.05 vs. the KA group, ^**^P < 0.01 vs. the KA group, # *P* < 0.05 vs. the KA+synbiotic group. KA, kainic acid.

The effects of modulating gut microbiota on the characteristics of spontaneous seizures in rats following SE were investigated in this study (Figure 1). After SE induction, treatment with prebiotics, probiotics, and synbiotics extended the latency period (prebiotics, *P* < 0.05; probiotics, *P* < 0.01; synbiotics, *P* < 0.01), decreased the frequency of seizures (prebiotics, *P* < 0.05; probiotics, *P* < 0.01; synbiotics, *P* < 0.01), and decreased the duration of seizures (prebiotics, *P* < 0.05; probiotics, *P* < 0.01; synbiotics, *P* < 0.01). In particular, the effect of synbiotics on the latency period and frequency of seizures was more obvious. Regarding the severity of seizures, treatment with synbiotics significantly decreased the scores for spontaneous seizures (*P* < 0.05).

### Modulating the gut microbiota improves cognitive deficit in epileptic rats

**Figure 2:**
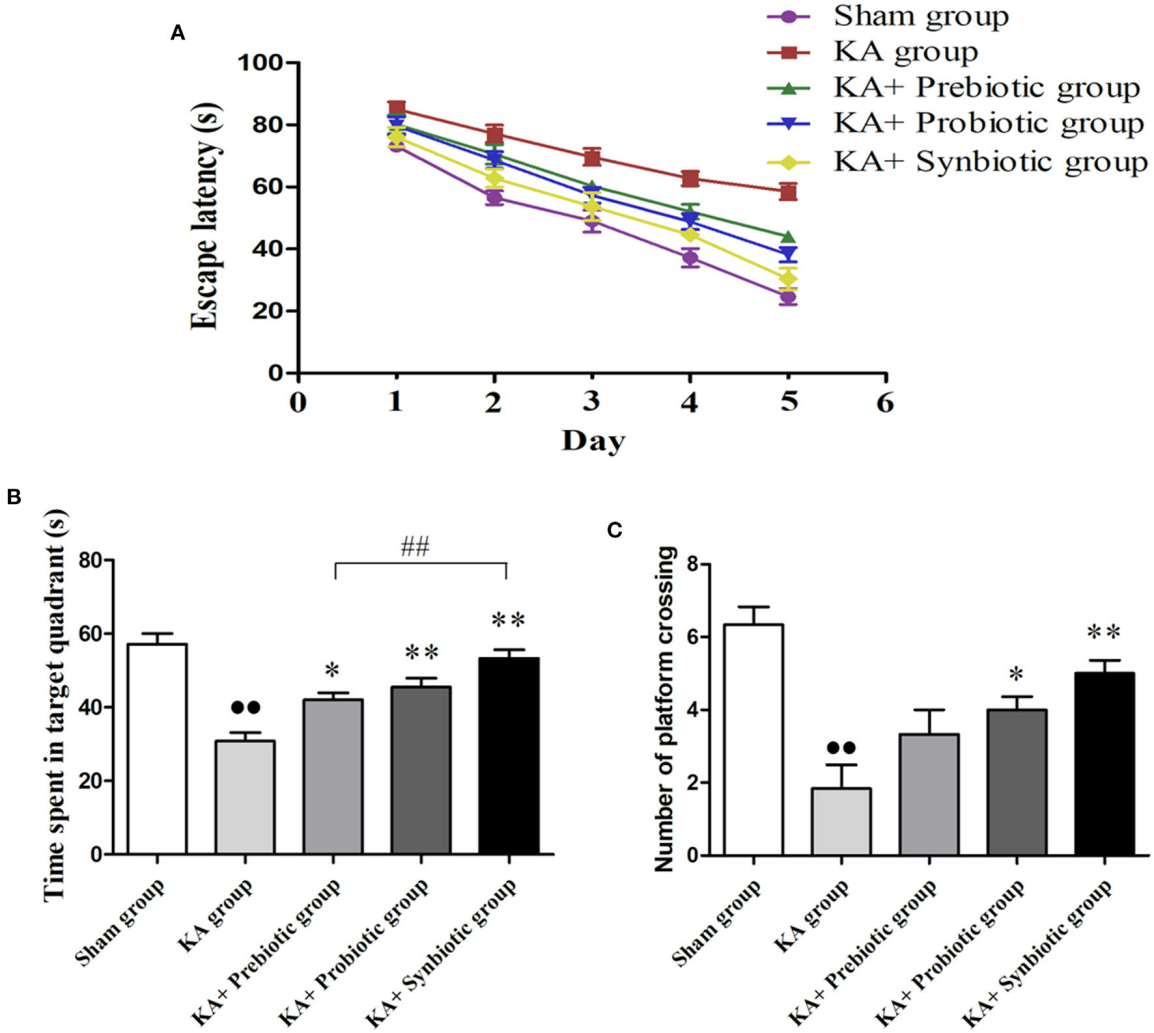
Effect of prebiotics, probiotics, and synbiotics on cognitive function in rats following status epilepticus induction. **(A)** Escape latency. **(B)** Time spent in the target quadrant. **(C)** Number of platform crossings. ∙∙ *P* < 0.01 vs. the Sham group, *P < 0.05 vs. the KA group, **P < 0.01 vs. the KA group, ## *P* < 0.01 vs. the KA+synbiotic group. KA, kainic acid.

The MWM test was used to evaluate the learning and memory abilities of the experimental rats. As shown in Figure 2, the escape latency of rats from the five groups gradually reduced during the five-day trial. On the fifth day, the escape latency of rats in the KA group was much longer than that in the sham group (*P* < 0.01); treatment with prebiotics (*P* < 0.01), probiotics (*P* < 0.01), or synbiotics (*P* < 0.01) significantly reduced escape latency (Figure 2A).

On the sixth day of the spatial probe test, rats in the KA group spent less time in the target quadrant (*P* < 0.01, Figure 2B) and crossed fewer platforms (*P* < 0.01, Figure 2C) compared with the sham group. Administration of prebiotics (*P* < 0.05), probiotics (*P* < 0.01), or synbiotics (*P* < 0.01) significantly increased the time spent in the target quadrant (Figure 2B). The number of platform crossings (Figure 2C) was significantly increased by treatment with probiotics (*P* < 0.05) or synbiotics (*P* < 0.01).

### Modulating the gut microbiota mitigates oxidative stress, DNA damage, and glutamate release following SE

**Figure 3:**
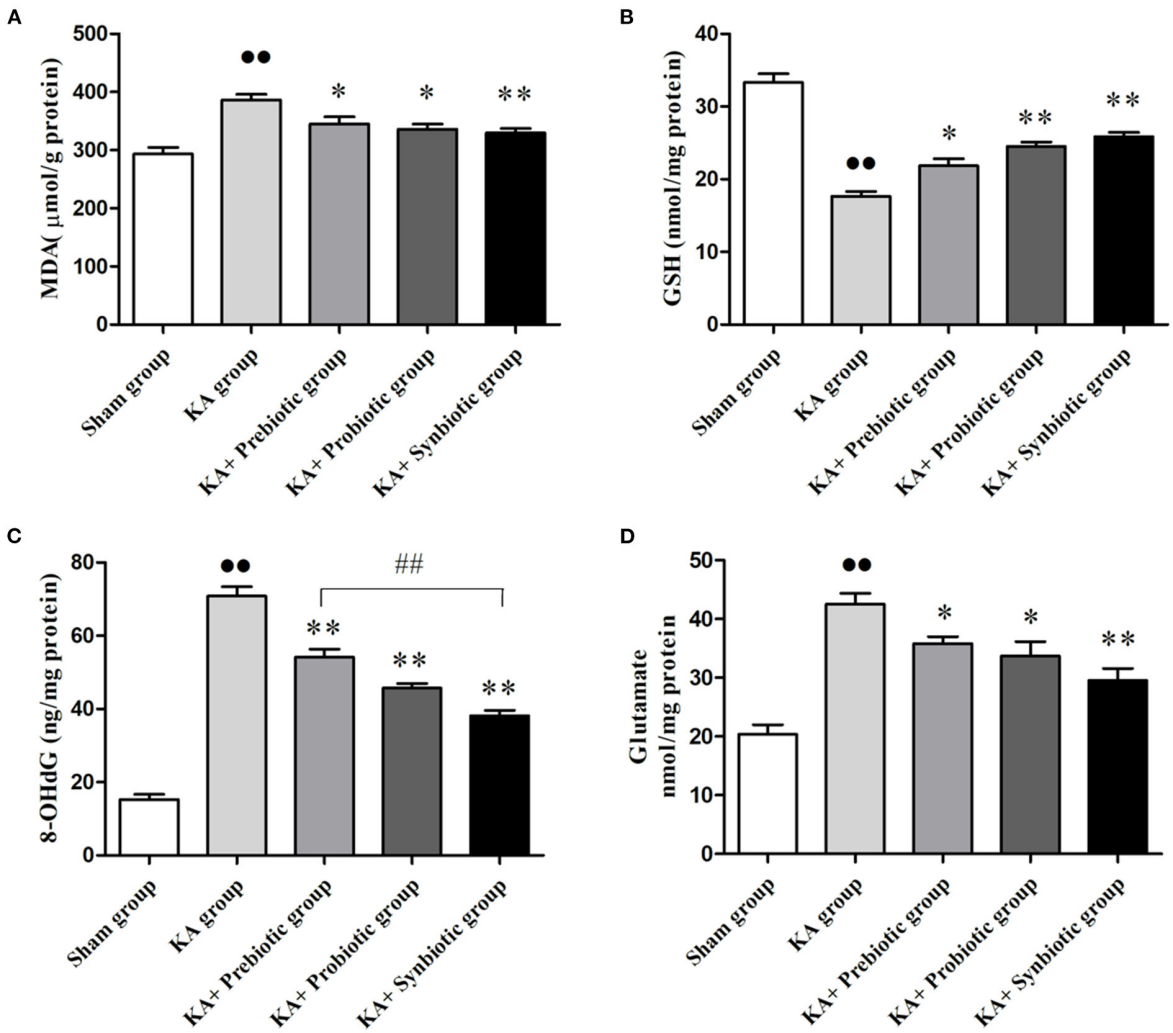
Treatment with prebiotics, probiotics, and synbiotics reduced the levels of MDA, 8-OHdG, and glutamate and increased the level of GSH in the hippocampus of rats after status epilepticus induction. **(A)** MDA. **(B)** GSH. **(C)** 8-OHdG. **(D)** Glutamate. ∙∙ *P* < 0.01 vs. the Sham group, ^*^P < 0.05 vs. the KA group, ^**^P < 0.01 vs. the KA group, ## *P* < 0.01 vs. the KA+synbiotic group. KA, kainic acid.

The role of modulating the gut microbiota in oxidative stress, DNA damage, and glutamate in rats following SE was evaluated. As shown in Figure 3, the rats in the KA group exhibited significantly higher levels of MDA (*P* < 0.01), 8-OHdG (*P* < 0.01), and glutamate (*P* < 0.01) and lower levels of GSH (*P* < 0.01) than those in the sham group. Administration of prebiotics, probiotics, and synbiotics significantly decreased the levels of MDA (prebiotics, *P* < 0.05; probiotics, *P* < 0.05; synbiotics, *P* < 0.01), 8-OHdG (prebiotics, *P* < 0.01; probiotics, *P* < 0.01; synbiotics, *P* < 0.01), and glutamate (prebiotics, *P* < 0.05; probiotics, *P* < 0.05; synbiotics, *P* < 0.01), and increased the levels of GSH (prebiotics, *P* < 0.05; probiotics, *P* < 0.01; synbiotics, *P* < 0.01) in KA-induced rats.

### Modulating the gut microbiota inhibits the activation of astrocytes and microglia and reduces inflammation following SE

**Figure 4:**
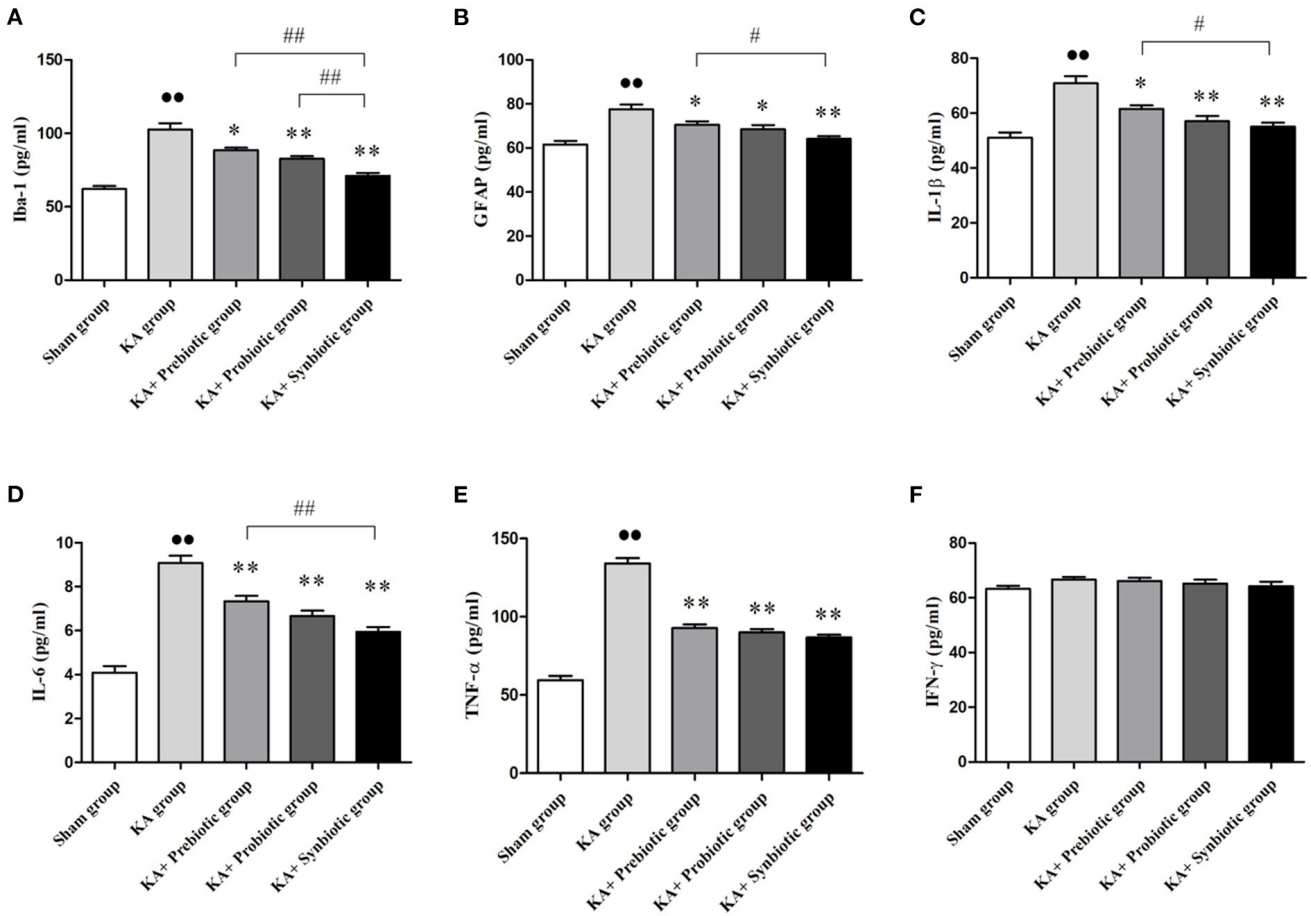
Treatment with prebiotics, probiotics, and synbiotics decreased the levels of Iba-1 and GFAP and inhibited the release of inflammatory cytokines such as IL-1 β, IL-6, and TNF- α. **(A)** Iba-1. **(B)** GFAP. **(C)** IL-1β. **(D)** IL-6. **(E)** TNF- α. **(F)** IFN- γ. ∙∙ *P* < 0.01 vs. the Sham group, *P < 0.05 vs. the KA group, **P < 0.01 vs. the KA group, ## *P* < 0.01 vs. the KA+synbiotic group. KA, kainic acid.

The role of modulating the gut microbiota on glial cells and inflammatory cytokines in rats following SE was also investigated, the results of which are shown in Figure 4. Rats in the KA group presented the activation of microglia (*P* < 0.01) and astrocytes (*P* < 0.01) and higher levels of IL-1β (*P* < 0.01), IL-6 (*P* < 0.01), and TNF-α (*P* < 0.01) compared with those in sham group. Treatment with prebiotics, probiotics, or synbiotics significantly inhibited the activation of microglia (prebiotics, *P* < 0.05; probiotics, *P* < 0.01; synbiotics, *P* < 0.01) and astrocytes (prebiotics, *P* < 0.05; probiotics, *P* < 0.05; synbiotics, *P* < 0.01) and reduced the levels of IL-1β (prebiotics, *P* < 0.05; probiotics, *P* < 0.01; synbiotics, *P* < 0.01), IL-6 (prebiotics, *P* < 0.01; probiotics, *P* < 0.01; synbiotics, *P* < 0.01), and TNF-α (prebiotics, *P* < 0.01; probiotics, *P* < 0.01; synbiotics, *P* < 0.01). No statistically significant difference was observed in IFN-γ levels between these groups.

## Discussion

This study found that modulating the gut microbiota had a positive impact on seizure control and cognitive deficits in KA-induced SE rats. Moreover, changes in oxidative stress parameters, DNA damage, glutamate release, activation of microglia and astrocytes, and levels of inflammatory cytokines in the hippocampal tissues were measured to study the role of modulating gut microbiota in their anti-seizure and cognition-enhancing effects.

Gut dysbiosis has gradually been recognized to be associated with epilepsy (22). Peng et al. studied alterations in gut microbiome composition between patients with drug-resistant epilepsy (DRE) and healthy individuals using 16S rDNA sequencing and found an increased abundance of rare flora in patients with DRE (8). In addition, those with four or more seizures per year showed lower levels of *Bifidobacteria* and *Lactobacillus* than those with fewer than four seizures per year (8). This study provides evidence that restoring the gut microbiota may be a novel therapeutic strategy for treating DRE. We used two bacterial strains, *Bifidobacterium* and *Lactobacillus*, as probiotics and inulin as a prebiotic, which were beneficial to the growth and activity of these probiotic strains. Our results showed that treatment with prebiotics, probiotics, and synbiotics ameliorated spontaneous seizures and cognitive deficits in KA-induced SE rats. As a matter of interest, synbiotics seemed to have a better curative effect than either prebiotics or probiotics alone.

Glial cell-mediated inflammation plays an important role in the development of epilepsy (23). A dysfunction in glial cells leads to the abnormal regulation of water, ions, and neurotransmitters, promoting hyperexcitability and hypersynchrony in the brain and increasing susceptibility to epilepsy (24). Uncontrolled glial-mediated immune reactions could cause a sustained inflammatory response and facilitate the development of epilepsy (24). Gut dysbiosis might be involved in the inflammatory mechanisms underlying epilepsy. Gut bacteria can affect neural networks by releasing neurotransmitters or their precursors, such as γ-aminobutyric acid (GABA) and glutamate (25, 26). The imbalance between excitatory and inhibitory neurotransmitters caused by gut dysbiosis contributes to the development of epilepsy. In addition, under normal conditions, gut bacteria can induce anti-inflammatory activity in the brain by stimulating the afferent neurons of the ENS *via* the vagus nerve. Disturbing normal bacterial abundance or function could disrupt the microbiota balance and lead to excess inflammation (27). Moreover, there may be a “leakage” in the integrity of the barrier system that participates in epileptogenesis, namely, the blood-brain barrier and intestinal mucosal barrier, which could also be affected by the gut microbiota. Gut dysbiosis may also induce excessive LPS production, which increases the permeability of the intestinal immune barrier, leading to epilepsy (27, 28). In the present study, we observed that modulating the gut microbiota *via* treatment with prebiotics, probiotics, and synbiotics effectively inhibited the inflammatory response and release of glutamate in the hippocampal tissues of rats following SE induction.

Oxidative stress is an important factor in the pathogenesis of epilepsy (29). Seizure activity can increase lipid peroxidation and decrease total antioxidant ability (30). The excessive generation of reactive oxygen species (ROS) can cause neuronal cell damage, which is associated with spontaneous seizures and cognitive deficits (29, 31). The gut microbiota may also influence the oxidative state in the central nervous system by interfering with the levels of ROS and the antioxidant system (32, 33). Abnormal changes in various metabolites, such as short-chain fatty acids, vitamins, and absorbable vitamins, can also regulate the oxidative state of the brain. These mechanisms have mostly been inferred from previous studies but still lack direct evidence. The present study confirmed that modulating the gut microbiota decreased lipid peroxidation, inhibited DNA damage, and enhanced total antioxidant ability, providing direct evidence that the gut microbiota affects the oxidative state during epilepsy.

## Conclusion

The present study demonstrated that modulating the gut microbiota by treatment with prebiotics, probiotics, and synbiotics exerts an anti-seizure effect and ameliorates cognitive impairment in KA-induced epileptic rats. Moreover, the results highlighted that modulating the gut microbiota inhibited oxidative stress and the inflammatory response in the hippocampus of epileptic rats, suggesting that modulating the gut microbiota could be a new therapeutic strategy to inhibit the development of epilepsy.

## Data availability statement

The original contributions presented in the study are included in the article/supplementary material, further inquiries can be directed to the corresponding author.

## Ethics statement

The animal study was reviewed and approved by Animal Studies Subcommittee of the Capital Medical University.

## Author contributions

XW wrote the draft of this manuscript. CY conducted the experiments. LY analyzed the data of this study. YZ revised the manuscript and gave the final approval. All authors contributed to the article and approved the submitted version.

## Conflict of interest

The authors declare that the research was conducted in the absence of any commercial or financial relationships that could be construed as a potential conflict of interest.

## Publisher's note

All claims expressed in this article are solely those of the authors and do not necessarily represent those of their affiliated organizations, or those of the publisher, the editors and the reviewers. Any product that may be evaluated in this article, or claim that may be made by its manufacturer, is not guaranteed or endorsed by the publisher.
